# Diagnosis and clinical management of severe acute respiratory syndrome Coronavirus 2 (SARS-CoV-2) infection: an operational recommendation of Peking Union Medical College Hospital (V2.0): Working Group of 2019 Novel Coronavirus, Peking Union Medical College Hospital

**DOI:** 10.1080/22221751.2020.1735265

**Published:** 2020-03-14

**Authors:** Taisheng Li

**Affiliations:** Department of Infectious Diseases, Peking Union Medical College Hospital, Chinese Academy of Medical Sciences & Peking Union Medical College, Beijing, People’s Republic of China

**Keywords:** Coronavirus disease 2019, SARS-CoV-2, pneumonia, diagnosis, treatment

## Abstract

Since December 2019, China has been experiencing an outbreak of a new infectious disease caused by severe acute respiratory syndrome coronavirus 2 (SARS-CoV-2). The clinical features include fever, coughing, shortness of breath, and inflammatory lung infiltration. China rapidly listed SARS-CoV-2-related pneumonia as a statutory infectious disease. To standardize the diagnosis and treatment of this new infectious disease, an operational recommendation for the diagnosis and management of SARS-CoV-2 infection is developed by Peking Union Medical College Hospital.

**Severe Acute Respiratory Syndrome Coronavirus 2** (SARS-CoV-2) is a novel type of coronavirus of β genus that leads to an emerging infectious disease with remarkable pulmonary involvement in China since December 2019. The clinical features include fever, dry cough, shortness of breath, normal or low levels of peripheral white blood cells, and inflammatory changes on chest X-ray. China has designated SARS-CoV-2 infected pneumonia as a statutory infectious disease. To standardize the clinical diagnosis and treatment, Peking Union Medical College Hospital (PUMCH) has established a working group and formulated the following operational recommendation regarding “Diagnosis and Clinical Management of Severe Acute Respiratory Syndrome Coronavirus 2 Infection” (V2.0).

## Protection requirements of medical personnel

### Selection of front-line personnel

Front-line medical personnel are qualified only after passing the physical examinations and professional training of SARS-CoV-2. Staff with the following conditions are exempt from SARS-CoV-2 related clinical/laboratory work, including pregnancy, age over 55 years old, a past history of chronic diseases such as chronic hepatitis, renal diseases, diabetes mellitus, autoimmune diseases and tumours. Individuals affected with acute fever should also be excluded from SARS-CoV-2 related work.

Baseline tests should be arranged including the complete blood count, urine analysis, biochemical tests, creatine kinase, and chest X-ray.

### Isolation and protection requirements

See “The guidelines for the diagnosis and treatment of severe acute respiratory syndrome coronavirus 2(SARS-CoV-2)infection (Pilot 3rd version)” published by the National Health Commission of the People’s Republic of China [1].

### Isolation and observation of medical personnel after close contact with SARS-CoV-2

1. Medical personnel in close contact with SARS-CoV-2 infected pneumonia patients should be relatively isolated, avoiding walking around and extensive contact with others.
2. Medical personnel should be isolated immediately and receive relevant examinations upon onset of fever, cough, shortness of breath and other symptoms.
3. When work in the SARS-CoV-2 infection ward is finished, nasopharyngeal or oropharyngeal swabs for SARS-CoV-2 and a complete blood count should be carried out. Those who have abnormal test results should undergo strict isolation and observation; while others will be generally isolated for observation and resume work after one week.

## Diagnosis and treatment of SARS-CoV-2 infected patients

### Screening criteria [1–2]

1. Epidemiological history: History of travel or residence in Hubei province within 2 weeks prior to the onset of illness, or contact with patients from Hubei province with fever and respiratory symptoms within 14 days prior to onset, or presented with clustering onset.
2. Acute onset of fever within 72 h without influenza-like symptoms, which could not attribute to other confirmed etiology.

### Diagnostic criteria

1. Supportive epidemiological history
2. Clinical manifestation: Fever; normal or low levels of white blood cells or decreased lymphocyte counts at onset. Chest radiology at early stage is characteristic of multiple small patchy shadows and interstitial changes, more prominent in the extrapulmonary bands. Multiple ground-glass opacities and infiltrations may develop bilaterally with disease progression, with possible consolidation in severe cases.
3. Diagnosis: SARS-CoV-2 nucleic acid positive in samples of sputum, pharynx swabs, and secretions of lower respiratory tract tested by real-time reverse-transcriptase–polymerase-chain reaction (rRT-PCR) assay.
4. For patients with acute fever (>37.5°C within 72 hours) and normal chest imaging, if the absolute count of peripheral lymphocytes is less than 0.8 × 10^9^/L, or the count of CD4^+^ and CD8^+^ T cells decreases significantly, isolation and close observation should be conducted at home even if the first SARS-CoV-2 nucleic acid test is negative. Repeat of rRT-PCR should be considered after 24 h, and a chest CT scan should be performed when necessary.

### Examination routines of SARS-CoV-2 infected patient

#### Screening cases on the day of visit

Nucleic acid examination of sputum or naso-/oropharyngeal swabs, complete blood count, urine analysis, arterial blood gas analysis, liver and kidney function, C-reactive protein (CRP), procalcitonin, creatine kinase plus myoglobin, coagulation, and chest CT should be performed. Inflammatory cytokines [such as interleukin (IL)-6, IL-10, and tumour necrosis factor (TNF)-α], TB lymphocyte subsets, and complement can be tested as appropriate [3–5].

#### Sequential examination of confirmed patients

1. Complete blood count, liver and kidney function, creatine kinase and myoglobin, coagulation function and CRP can be checked on the 3rd, 5th and 7th days after admission and on discharge according to the disease status. PCT and TB lymphocyte subsets can be repeated on days 5–7 if feasible [3–5].
2. The chest X-ray or CT scan should be re-examined 1–2 days after the admission, and the time for subsequent re-examination depends on the disease status, no longer than 5 days.
3. Complete blood count, chest X-ray, liver and kidney function, and all abnormal examinations on admission should be re-examined before discharge except for referrals.

### Place of treatment according to the severity of the disease

All cases with screening indications are subject to on-site medical isolation (single-room isolation), and once diagnosed, should be transferred to a designated hospital.

#### Severe type

According to the definition of the National Health Commission [1], patients in accordance with one of the following standards should be hospitalized and transferred to Beijing designated medical institution as soon as possible; (1) respiratory rate increased (≥30 per min) or dyspnoea; (2) oxygen saturation ≤ 95% when breathing ambient air, or arterial oxygen tension (PaO₂) over inspiratory oxygen fraction (FIO₂) of less than 300 mm Hg (1 mm Hg equals to 0.133 kPa); (3) lung imaging indicating multilobular lesions or progression of lesions over 50% within 48 h; (4) quick sequential organ failure assessment (qSOFA) score ≥2; (5) community-acquired pneumonia-65 (CURB-65) score ≥ 1; (6) combined pneumothorax; (7) other clinical conditions that require hospitalization.

#### Critically ill type

According to the definition of the National Health Commission [1], patients in accordance with respiratory failure, septic shock or other organ failure should be transferred to intensive care unit immediately and to designated medical institution as soon as possible when feasible.

### Treatment

#### General treatment

Patients should be kept in bed and closely monitored for vital signs and levels of oxygen saturation. Supportive treatment should be ensured, including enough supply of energy and fluid, maintenance of electrolyte and acid–base homeostasis.

#### Oxygen therapy

Patients with hypoxemia should be given oxygen therapy immediately and maintain a blood oxygen saturation level to no less than 90% in man and non-pregnant women, and between 92% and 95% in pregnant women.

##### Choice of oxygen therapy

Patients with mild hypoxemia should be put on nasal cannula, 5 L/min. If the patient is getting worse, high flow nasal cannula should be considered, starting with 20 L/min and increasing to 50–60 L/min gradually. Fraction of oxygen should be adjusted according to oxygen saturation.

##### The way of respiratory support

Non-invasive ventilation is only considered for patients who can tolerate. For the patients requiring invasive ventilation, endotracheal intubation should be performed by experienced physician with personal protective equipment.

We recommend protective ventilation strategy for acute respiratory distress syndrome (ARDS). For those patients with most severe ARDS, extracorporeal membrane oxygenation or prone position is recommended. Interventions should be implemented to prevent complications associated with critical illness. Standard precautions should always be routinely applied in all areas of healthcare facilities.

#### Antiviral treatment

Currently, there is no evidence to support the effectiveness of existing antiviral drugs against SARS-CoV-2. Lopinavir/ritonavir can be used when appropriate, 2 tablets, twice daily for 14 days.

#### Glucocorticoid therapy

Severe patients could receive glucocorticoid at early stage, e.g. intravenous methylprednisolone 40–80 mg, once daily for 5 days, and the course of treatment can be prolonged according to the clinical condition and radiological manifestations.

#### Intravenous immunoglobulin

Early intravenous infusion of human immunoglobulin is recommended for critically ill patients, based on their clinical condition, at 0.25–0.5 g/(kg·d), for 3–5 days [6–7].

#### Empirical antimicrobial therapy

If bacterial infection is suspected according to the patient's clinical and imaging findings, patients of mild type can take oral antibiotics for community-acquired pneumonia, such as second-generation cephalosporins or fluoroquinolones. As regards patients of severe type, all possible pathogens should be covered when necessary.

## Protection and transfer

1. Once the critically ill patients are diagnosed and endotracheal intubation is anticipated, they should be immediately transferred to the ICU ward with negative pressure. All the procedures should be carried out in accordance with the requirements of comprehensive protection.
2. Oxygen storage mask is used to supply oxygen above 15 L/min during transport, during which complete filling of the oxygen storage airbag should be ensured.
3. Endotracheal intubation should be induced by standard rapid procedure, and muscle relaxants should be used as much as possible to avoid droplet transmission caused by choking.
4. Reusable items such as goggles should be disinfected after intubation before taken out of the negative pressure ward.
5. Patients with intubation should use closed endotracheal suction to avoid airborne transmission caused by ventilator airflow.
6. Under certain circumstances when the ventilator must be disconnected for operation, the standby function of the ventilator should be set to avoid airborne transmission caused by ventilator airflow. Once the standby function is not available, the Y-tube port of the ventilator should be blocked to avoid air spread.

## Criteria of isolation release and discharge [1]

Patients could be discharged or transferred to the other departments for comorbidities, when the body temperature returned to normal for more than 3 days, the respiratory symptoms significantly improved, the pulmonary lesions markedly absorbed, and respiratory nucleic acid is tested negative for SARS-CoV-2 for two consecutive times at least 1 day apart.

**Wrote by**

Taisheng Li (Department of Infectious Diseases, Peking Union Medical College Hospital).

Wei Cao (Department of Infectious Diseases, Peking Union Medical College Hospital).

Li Weng (Department of Internal Medicine ICU, Peking Union Medical College Hospital).

Hongwei Fan (Department of Infectious Diseases, Peking Union Medical College Hospital).

Juhong Shi (Department of Respiratory and Critical Medicine, Peking Union Medical College Hospital).

**Experts participating in the discussion (sorted by family name of Chinese Phonetic Alphabet)**

Wenzhao Chai, Bin Du, Na Guo, Ding Han, Yang Han, Xiaoyun Hu, Yang Jiao, Zhengyu Jin, Zhengyin Liu, Yun Long, Xiaojun Ma, Huizhen Wang, Mengzhao Wang, Xinming Wu, Yingchun Xu, Wenbing Xu, Bo Zhang, Fengchun Zhang, Shengjie Zhang, Huadong Zhu
